# Polymer-Stabilized Elemental Boron Nanoparticles for Boron Neutron Capture Therapy: Initial Irradiation Experiments

**DOI:** 10.3390/pharmaceutics14040761

**Published:** 2022-03-31

**Authors:** Alexander Zaboronok, Polina Khaptakhanova, Sergey Uspenskii, Raman Bekarevich, Ludmila Mechetina, Olga Volkova, Bryan J. Mathis, Vladimir Kanygin, Eiichi Ishikawa, Anna Kasatova, Dmitrii Kasatov, Ivan Shchudlo, Tatiana Sycheva, Sergey Taskaev, Akira Matsumura

**Affiliations:** 1 Department of Neurosurgery, Faculty of Medicine, University of Tsukuba, 1-1-1 Tennodai, Tsukuba 305-8575, Japan; e-ishikawa@md.tsukuba.ac.jp (E.I.); matsumura.akira.ft@alumni.tsukuba.ac.jp (A.M.); 2 Laboratory of Medical and Biological Problems of BNCT, Department of Physics, Novosibirsk State University, 1 Pirogov Street, 630090 Novosibirsk, Russia; kanigin@mail.ru; 3 Enikolopov Institute of Synthetic Polymeric Materials, Russian Academy of Sciences, 70, Profsoyuznaya Street, 117393 Moscow, Russia; polinakhap@yandex.ru (P.K.); s.a.uspenskii@mail.ru (S.U.); 4 The Centre for Research on Adaptive Nanostructures and Nanodevices (CRANN), Advanced Microscopy Laboratory, Trinity College Dublin, The University of Dublin, D02 W272 Dublin, Ireland; raman.bekarevich@tcd.ie; 5 Research Center for Advanced Measurement and Characterization, National Institute for Materials Science, 1-2-1 Sengen, Tsukuba 305-0047, Japan; 6 Laboratory of Immunogenetics, Institute of Molecular and Cellular Biology, Novosibirsk, 8/2 Lavrentieva, 630090 Novosibirsk, Russia; lucie@mcb.nsc.ru (L.M.); volkova@mcb.nsc.ru (O.V.); 7 International Medical Center, University of Tsukuba Hospital, 2-1-1 Amakubo, Tsukuba 305-8576, Japan; bmathis@md.tsukuba.ac.jp; 8 Budker Institute of Nuclear Physics, Siberian Branch of Russian Academy of Sciences, 11 Lavrentieva, 630090 Novosibirsk, Russia; yarullinaai@yahoo.com (A.K.); kasatovd@gmail.com (D.K.); i-vanshch@yandex.ru (I.S.); sychevatatyanav@gmail.com (T.S.); taskaev@inp.nsk.su (S.T.); 9 Laboratory of BNCT, Department of Physics, Novosibirsk State University, 1 Pirogov Street, 630090 Novosibirsk, Russia

**Keywords:** elemental boron nanoparticles, hydroxyethylcellulose, polymer stabilization, boron neutron capture therapy, accelerator-based neutron source

## Abstract

Sufficient boron-10 isotope (^10^B) accumulation by tumor cells is one of the main requirements for successful boron neutron capture therapy (BNCT). The inability of the clinically registered ^10^B-containing borophenylalanine (BPA) to maintain a high boron tumor concentration during neutron irradiation after a single injection has been partially solved by its continuous infusion; however, its lack of persistence has driven the development of new compounds that overcome the imperfections of BPA. We propose using elemental boron nanoparticles (eBNPs) synthesized by cascade ultrasonic dispersion and destruction of elemental boron microparticles and stabilized with hydroxyethylcellulose (HEC) as a core component of a novel boron drug for BNCT. These HEC particles are stable in aqueous media and show no apparent influence on U251, U87, and T98G human glioma cell proliferation without neutron beam irradiation. In BNCT experiments, cells incubated with eBNPs or BPA at an equivalent concentration of 40 µg ^10^B/mL for 24 h or control cells without boron were irradiated at an accelerator-based neutron source with a total fluence of thermal and epithermal neutrons of 2.685, 5.370, or 8.055 × 10^12^/cm^2^. The eBNPs significantly reduced colony-forming capacity in all studied cells during BNCT compared to BPA, verified by cell-survival curves fit to the linear-quadratic model and calculated radiobiological parameters, though the effect of both compounds differed depending on the cell line. The results of our study warrant further tumor targeting-oriented modifications of synthesized nanoparticles and subsequent in vivo BNCT experiments.

## 1. Introduction

Boron neutron capture therapy (BNCT) is an adjuvant radiotherapy method that requires sufficient boron-10 (^10^B) concentration in tumor tissue (≥20 µg ^10^B/g) and further neutron irradiation of the tumor area, resulting in the elimination of malignant cells by an intracellular nuclear decay reaction [1,2,3,4]. ^10^B-containing sodium borocaptate (BSH) and borophenylalanine (BPA), which have been widely used in preclinical and clinical BNCT experiments, have certain drawbacks that prevent them from becoming ideal drugs for BNCT [4,5]. BSH lacks active tumor targeting and cannot penetrate the blood–brain barrier, whereas BPA is washed out from tumor cells over time, necessitating continuous infusion to maintain an effective ^10^B concentration during neutron beam irradiation [6]. Motivated by these BSH and BPA limitations, various research groups have developed complex compounds and boron-containing liposomes aimed at more efficient boron delivery [7,8,9,10,11,12,13,14,15,16,17,18,19] and have performed appropriate computer simulations [20]. However, none of these compounds has reached clinical application.

Meanwhile, on 25 March 2020, BPA in the form of borofalan (Steboronine ^®^, Stella Pharma, Co. Ltd., Osaka, Japan) was approved for clinical use in BNCT for the treatment of head and neck cancer after clinical trials at the cyclotron-based accelerator constructed by Sumitomo Heavy Industries (Tokyo, Japan) [21,22]. Although this is a giant leap toward developing clinical BNCT in Japan (and worldwide), no new compound has yet been approved, and the limitations of BPA remain.

Modern nanotechnology permits the synthesis of a wide variety of nanoparticles with antitumor activity, such as self-assembled peptide-based supraparticles [23], reactive oxygen species-generating amine-functionalized magnetic nanoparticles (with the pH-responsive release of a chemotherapeutic agent) [24], ionically crosslinked complex gels loaded with active compound-containing vesicles for transdermal drug delivery [25], and green synthesized Ag and Mg dual-doped ZnO nanoparticles showing toxicity against tumor cells [26].

Delivering more boron to tumor cells can be solved using boron nanoparticles, which can carry thousands of boron atoms per particle and sequester in tumor cells longer due to differences in accumulation mechanisms [27,28,29,30]. The synthesis of boron nitride and boron carbide nanoparticles in related biological experiments has been reported [27,31,32,33,34,35]; however, these nanoparticles contain a significant amount of nitrogen or carbon that do not participate in neutron capture reactions. Borophenes have also been proposed as carriers of large amounts of boron atoms for BNCT [36], and, compared to boron nitride and boron carbide nanoparticles, they do not contain additional non-capture elements. However, unlike nanoparticles, for which delivery to tumor cells has been studied for decades, the interaction of borophenes with biological systems remains a subject for further research.

We recently proposed an original and relatively simple method of cascade ultrasonic dispersion/destruction of elemental boron microparticles in an aqueous medium to produce elemental boron nanoparticles (eBNPs) containing only boron without other elements [37,38]. Bare nanoparticles lack active tumor targeting and aggregate in aqueous solutions over time, forming clusters that make solutions unsuitable for further use in biological experiments. Therefore, we focused on finding a substance that would stabilize the stock solution, reduce the potential toxicity of nanoparticles, and act as an intermediate agent linking nanoparticles to tumor-targeting molecules.

As drug delivery systems based on polymeric materials can improve the pharmacological and therapeutic properties of drugs by controlling their pharmacokinetics and biodistribution [39,40], we searched for a suitable polymeric stabilizer with both a high nanoparticle loading capacity capable of maintaining effective boron concentrations in tumor cells over time and reactive functional groups for biomolecular vector attachment. Among the wide range of biocompatible and biodegradable polymers used to develop drug delivery systems, including biopolyethers and polyamino acids, polysaccharides are more suitable for encapsulating hydrophobic drugs [40]. A cellulose derivative, hydroxyethylcellulose (HEC), was approved by the U.S. Food and Drug Administration (FDA) for use in drug stabilization formulations, antigens, and vaccines (proteins, peptides, mRNA, and DNA [41]), and meets the requirements of the National Formulary (NF), European Pharmacopoeia (Ph. Eur./EP), and Japanese Pharmacopoeia (JPE) [42]. In addition, HEC is often used with hydrophobic drugs in various commercial products, such as capsules to improve drug dissolution for controlled delivery in oral medications (hydrophilization) or in eye drops for more efficient delivery of the active ingredient without undesirable carrier effects [43]. Negatively charged functional groups on the macromolecules of cellulose ethers provide a good platform for combination with various positively charged materials, such as metallic and nonmetallic nanoparticles.

Thus, in our study, we used elemental boron nanoparticles, or eBNPs, produced from ^10^B microparticles in an aqueous solution and stabilized with HEC. We hypothesized that our polymer-stabilized, ^10^B-containing eBNPs could significantly reduce the colony-forming capacity of tumor cells after neutron beam irradiation and become a promising core compound for the further development of BNCT-related boron drugs.

Here, we report the results of the initial in vitro BNCT experiments using our newly synthesized eBNPs accumulated in tumor cells and irradiated at an accelerator-based neutron source, comparing the effects of eBNPs to BPA. We focused on evaluating the hypothetical ability of eBNPs to remain in tumor cells during neutron irradiation after placing nanoparticle-containing cells in fresh boron-free medium, which should help to overcome the problem of washout and lead to more effective tumor growth suppression. We are the first to propose elemental boron nanoparticle synthesis using cascade ultrasonic dispersion/destruction of elemental boron microparticles and perform in vitro irradiation experiments using these eBNPs at a prototype accelerator for clinical BNCT.

## 2. Materials and Methods

### 2.1. Elemental Boron Nanoparticles (eBNPs)

Elemental boron nanoparticles were synthesized in two main steps—(1) cavitation dispersion of 0.5–4 μm amorphous boron particles with a mass fraction of boron ≥99.6% (National High Technology Centre, Tbilisi, Georgia) in an aqueous dispersion medium at 80 °C followed, by two-step cascade fractionation [37,38]. The dispersion was carried out for eight hours using an ultrasonic generator I-6/03-0/6 with a titanium alloy submersible probe set to an output power of 0.63 kW (Inlab Ltd., St. Petersburg, Russia).

The obtained dispersion of boron nanoparticles was studied using an aberration-corrected transmission electron microscope (TEM) with a cold field-emission gun operated at 200 kV (JEOL JEM ARM200F, JEOL Ltd., Tokyo, Japan), dynamic light scattering (DLS, Zetatrac, Microtrac MRB, York, PA, USA), and X-ray diffraction analysis (XRD, Bruker D8 Advance Diffractometer, Bruker Inc., Billerica, MA, USA). These eBNPs were further exposed to ultrasound for 5 min and stabilized with 0.3% hydroxyethylcellulose (HEC, 1000 kDa, Ashland Inc., Wilmington, DE, USA) aqueous solution by continuous stirring. The dynamic viscosity of a 0.3% HEC solution was measured by a vibrating viscometer (SV-10A, A&D, Tokyo, Japan) and equaled 4.5 mPa∙s. The stability of the obtained colloidal solution (particle aggregation with their cluster size changes over time and zeta potentials) was further studied by DLS.

The chemical composition of eBNPs can be described by the formula

(-C_5_H_5_O(OH)_2_-)*_m_*CH_2_OC_2_H_4_O----B*_n_*,

where *m* is the number of HEC molecules attached to the nanoparticle, and *n* is the number of boron atoms, which varies depending on the size of the boron core and equals approximately 12,000 to 50,000 for boron particle sizes of 3 to 50 nm, respectively.

**Figure 1:**
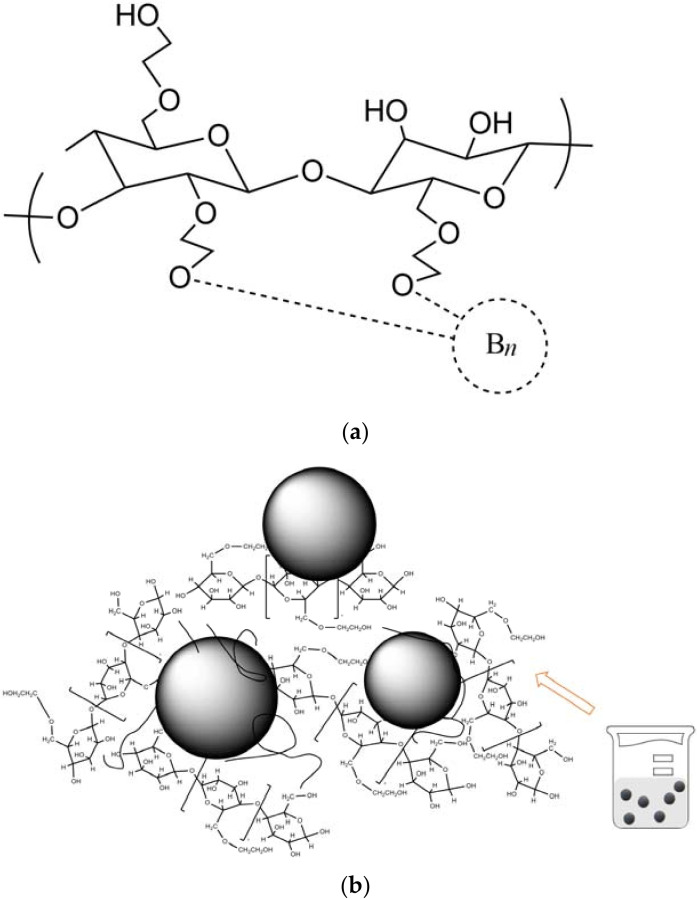
(**a**) Spatial interaction of boron and HEC. *n* = number of boron atoms in the particle. (**b**) Interaction of eBNPs with HEC in an aqueous solution (schematically). The formulas were drawn using ChemBioDraw Ultra software Version 14.0 (PerkinElmer, Inc., Waltham, MA, USA).

The spatial interaction of boron and HEC and the schematic configuration of eBNP and HEC complexes in an aqueous solution are presented in Figure 1.

### 2.2. Boronophenylalanine (BPA)

BPA (p-boronophenylalanine, ≥99.6% ^10^B) was purchased from Katchem Co., Ltd. (Prague, Czech Republic). The BPA-fructose solution was prepared as described previously [44,45]. In short, BPA (500 mg) and fructose (1100 mg) were mixed in 15 mL of Milli-Q water and 2.7 mL of 1 M NaOH solution with further neutralization by HCl to pH = 7.2. The resulting stock solution containing 1100 µg of ^10^B per ml was diluted to therapeutic concentrations and added to the cell-containing media.

### 2.3. Human Glioma Cell Lines

T98G, U87, and U251 human glioma cell lines were purchased from the Institute of Cytology at the Russian Academy of Sciences (St. Petersburg, Russia). The cells were cultured in Iscove’s Modified Dulbecco’s Medium (IMDM; SIGMA 17633 with L-glutamine and 25 mH HEPES, without sodium bicarbonate, Sigma-Aldrich, St. Louis, MO, USA) with 10% fetal bovine serum (FBS, Thermo Scientific HyClone SV30160.03 HyClone UK Ltd., Leicestershire, UK) and 1% Antibiotic Antimycotic Solution (Sigma-Aldrich, St. Louis, MO, USA) and maintained in an incubator at 37 °C in 5% CO_2_ atmosphere.

### 2.4. Cell Proliferation Assay

To assess the compound cytotoxicity, cellular proliferation after incubation with nanoparticles was assessed using MTS assay (Cell Titer 96^®^ Aqueous One Solution, Promega Corporation, Madison, WI, USA) [46,47], modified as described previously [48]. The cells from each line were placed in 96-well plates (Falcon^®^, Corning, Inc., Corning, NY, USA) in amounts of 4 × 10^4^ in 100 μL of medium per well and incubated for 24 h. Then, the medium was replaced with fresh culture medium containing eBNPs (0–250 µg ^10^B/mL) and the cells were further incubated for 24 h. After that, the medium with eBNPs was removed and the cells were washed with PBS. Next, 2 mL of 3-(4,5-dimethylthiazol-2-yl)-5-(3-carboxymethoxyphenyl)-2-(4-sulfophenyl)-2H-tetrazolium (MTS) solution with PMS and 10 mL of MEM were mixed, and 100 µL of this mixture was added to the cells in each well. Finally, the samples were incubated for 2 h and analyzed with a Bio-Rad Model 2550 EIA plate reader (Bio-Rad Inc., Hercules, CA, USA) with light absorption at 490 nm. Cell proliferation efficiency is presented as a ratio compared to untreated cells incubated without eBNPs. The long-term influence of BPA and eBNPs on cellular proliferation are shown as differences in cell plating (colony-forming) efficiency compared to untreated cells after 14 day-incubation.

### 2.5. Irradiation Experiments

After 24-h incubation with eBNPs or BPA (40 µg of ^10^B/mL), the cells were washed with phosphate-buffered saline (PBS (-), Fujifilm Wako Pure Chemical Corporation, Osaka, Japan), trypsinized with 0.05% trypsin–ethylenediaminetetraacetic acid (trypsin-EDTA, Nacalai Tesque, Inc., Kyoto, Japan), counted using an Improved Neubauer cell-counting chamber (NanoEnTek, Inc., Waltham, MA, USA), and 10^6^ of cells were placed in 1 mL plastic vials (Sumilon^®^, Sumitomo Bakelite Co., Ltd., Tokyo, Japan) in fresh culture medium without boron to allow for natural washout. Untreated, unirradiated, or irradiated cells were used as controls where appropriate.

Neutron beam irradiation was performed at a neutron source based on a vacuum-insulated tandem accelerator and lithium target constructed at the Budker Institute of Nuclear Physics (Novosibirsk, Russia) [49,50,51], a clinical BNCT accelerator prototype of the facility manufactured by TAE Life Sciences, Inc. (Foothill Ranch, CA, USA) [52]. The accelerator was operated at a proton current of 1.725–1.812 mA and an energy of 2.032 MeV. Based on the irradiation settings and previously experimentally studied spatial distribution of the beam components [53], the irradiation fluences were calculated by the Monte Carlo method using an NMC code developed at the Nuclear Safety Institute of the Russian Academy of Sciences (IBRAE RAS) [54]. The characteristics of the beam, its contamination with fast neutrons and photons, and the neutron spectra were similar to those described previously [51,53,55,56]. One milliampere-hour resulted in the following fluences: thermal neutrons—2.608 × 10^12^/cm^2^, epithermal neutrons—7.696 × 10^10^/cm^2^, fast neutrons—6.118 × 10^10^/cm^2^, and photons—7.201 × 10^11^/cm^2^, with the absorbed doses as follows: fast neutrons—0.314 Gy, photons—2.997 Gy. Photon spectra were presented with the following energies: 0.020—0.100 MeV (13.822%, superficial X-rays, which could mainly affect the cells [48,57]), 0.100—0.500 MeV (47.064%), 0.500—2.000 MeV (5.023%), 2.000—2.510 MeV (32.521%), and 2.500—12.600 MeV (1.57%).

**Figure 2:**
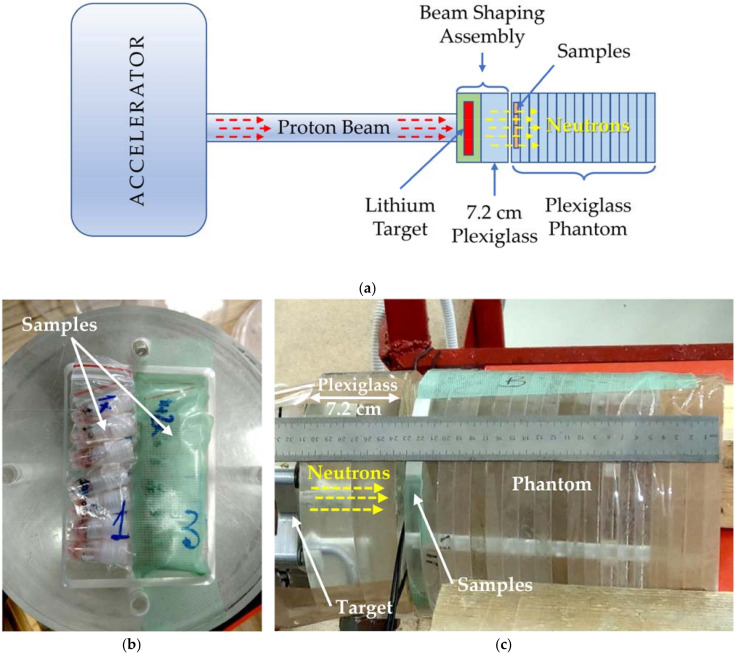
Irradiation settings. Schematic illustration of BNCT accelerator components and irradiated samples (**a**). Cells in vials were placed in the first layer of the plexiglass phantom (**b**). The phantom was oriented horizontally with a 7.2 cm plastic layer between the neutron-producing target and the cells (**c**).

The samples were placed vertically in the installation with a horizontally oriented neutron beam. The irradiation settings are shown in Figure 2.

Since the BNCT effect was assumed to be associated with thermal (which dominated here) and epithermal neutron fluences, their sum was used to calculate radiobiological parameters. The samples were irradiated with the total (thermal and epithermal) neutron fluences of 2.685, 5.370, or 8.055 × 10^12^/cm^2^, corresponding to one, two, and three milliampere-hours (mAh), respectively. The samples were respectively marked as 1×, 2×, and 3× fluence-irradiated samples. The total neutron generation with sample irradiation lasted approximately three hours in each of the three independent experiments. The initial setting included 1× and 3× samples and, after gaining the fluence of 2.685 × 10^12^/cm^2^, or 1×, the corresponding 1× samples were removed from the phantom and replaced with 2× samples that were further irradiated with 2× fluence together with 3× samples, which received the total maximum dose. Thus, the samples irradiated with the maximum 8.055 × 10^12^/cm^2^ fluence were in the phantom for the entire time of irradiation.

### 2.6. Colony-Forming Assays (CF-Assays)

After irradiation, the samples were transferred to the cell laboratory and the cells were extracted from the vials, washed, counted, diluted, and seeded into 6 cm round plastic dishes. Depending on the neutron fluence, 200 to 2000 cells per dish were empirically seeded. Fourteen days after irradiation, the cells were washed with PBS, fixed with glutaraldehyde, and stained with crystal violet. The dishes were scanned and the colonies (≥50 cells) were counted [48,58].

### 2.7. Radiobiological Parameters Calculation

Based on the number of colonies, cell survival curves were plotted as a function of neutron fluence and fit to the linear-quadratic (LQ) model using the equation $SF=e^{-\left(\alpha C+\beta C^{2}\right)}$, where SF was the surviving fraction [59,60]. Radiobiological parameters *α* (*alpha*) and *β* (*beta*) were calculated from the cell survival curve fits in Microsoft Excel with the SOLVER add-on (Microsoft, Inc., Redmond, WA, USA) [58,61]. Differences in survival fractions between the cells irradiated with eBNPs, BPA, or without boron were evaluated by comparing the corresponding areas under the fitted curves (AUCs), which corresponded to the definite integrals of the LQ function with the fluence (*F*) range as a function of *x* [58,61]:

$$AUC=\int_{0}^{Fmax}\exp\left(-\alpha F-\beta F^{2}\right)dF.$$

*F*_10_, the neutron fluence required to eliminate 90% of the tumor cells, leaving 10% of surviving colonies, was calculated as follows:

$$\alpha F+\beta F^{2}+ln\left(SF\right)=0,$$

where *F* was the fluence (×10^12^/cm^2^) calculated using the following equation:

$$F=\frac{-\alpha \pm \sqrt{\alpha^{2}-4\beta ln\left(SF\right)}}{2\beta },$$

with positive values representing *F*.

### 2.8. Statistical Analysis

The obtained data were within one standard deviation (SDs) of the means, if not otherwise indicated. One-way analysis of variance (ANOVA) was used to verify the differences among the obtained parameters, with *p*-values ≤ 0.05 indicating statistical significance [62].

## 3. Results

### 3.1. Nanoparticle Characteristics

**Figure 3:**
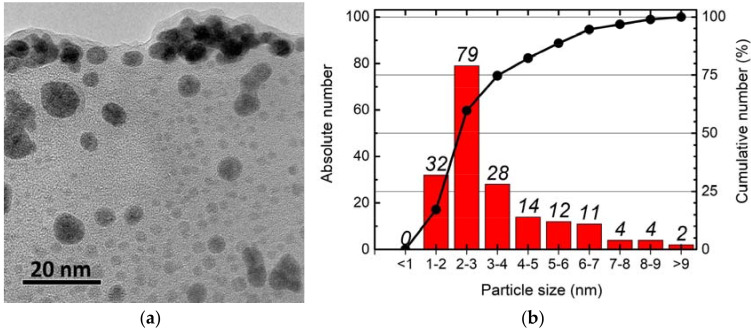
eBNPs visualized by TEM (magnification × 1.5 million) (**a**) and nanoparticle size distribution (**b**).

On transmission electron microscope (TEM) images, we observed slightly elongated, spherical eBNPs, sized 1–12 nm, with a major fraction of 1–4 nm nanoparticles (Figure 3).

The X-ray diffraction analysis showed peaks characterizing amorphous boron both before and after ultrasonic processing, indicating the amorphous composition of the obtained nanoparticles which was described in our previous report [37]. Although the newly synthesized nanoparticles differed significantly in size and formed larger clusters in aqueous solution over time, the formation of complexes with HEC resulted in more homogeneous particles of a larger size and increased solution stability over time (Table 1).

### 3.2. Nanoparticle Cytotoxicity

**Figure 4:**
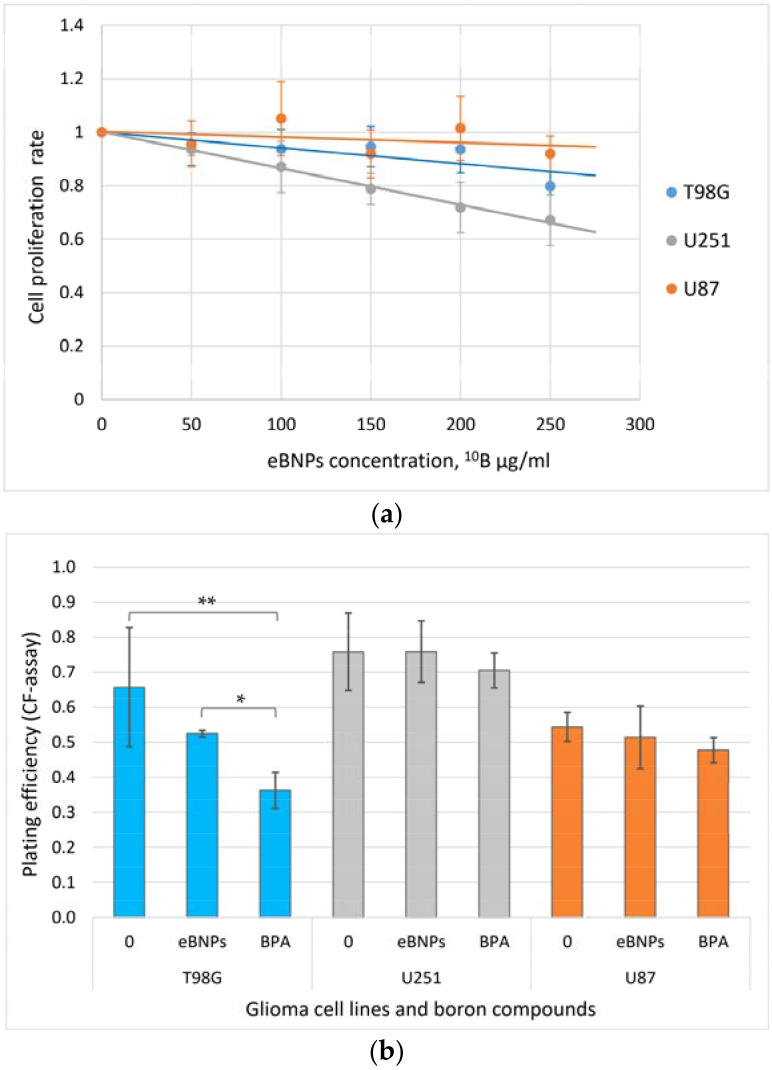
(**a**) Cancer cell survival after 24 h of incubation with nanoparticles evaluated by MTS assay (**b**). Cell plating efficiency 14 days after a 24-h incubation with 40 µg of ^10^B/mL of eBNPs or BPA, or without boron compounds analyzed by CF-assay; intergroup comparison by one-way ANOVA, * *p =* 0.003, ** *p =* 0.021, no significant difference in other groups (*p* > 0.05).

The short- and long-term influence of eBNPs on cell proliferation representing compound cytotoxicity without irradiation was analyzed in two series of experiments (Figure 4). The nanoparticles did not significantly affect cellular proliferation in the range of the minimum clinically relevant therapeutic concentration of 20 µg/mL, with a further slight increase in the suppression of cellular proliferation at concentrations exceeding the minimum therapeutic range (50–250 µg/mL). U251 cells showed a more significant response to the increased concentration of boron nanoparticles than the other two cell lines. Cell survival rates as a function of the eBNP concentration for each cell line are shown in Figure 4a.

After 14 days of incubation, the plating efficiency did not differ significantly between the groups in the U251 and U87 cells. In the T98G cells, BPA showed significantly higher cellular proliferation inhibition compared to eBNPs and the untreated control, while the differences in the other cell lines did not reach significance (Figure 4b).

### 3.3. Glioma Cell Survival after BNCT

**Figure 5:**
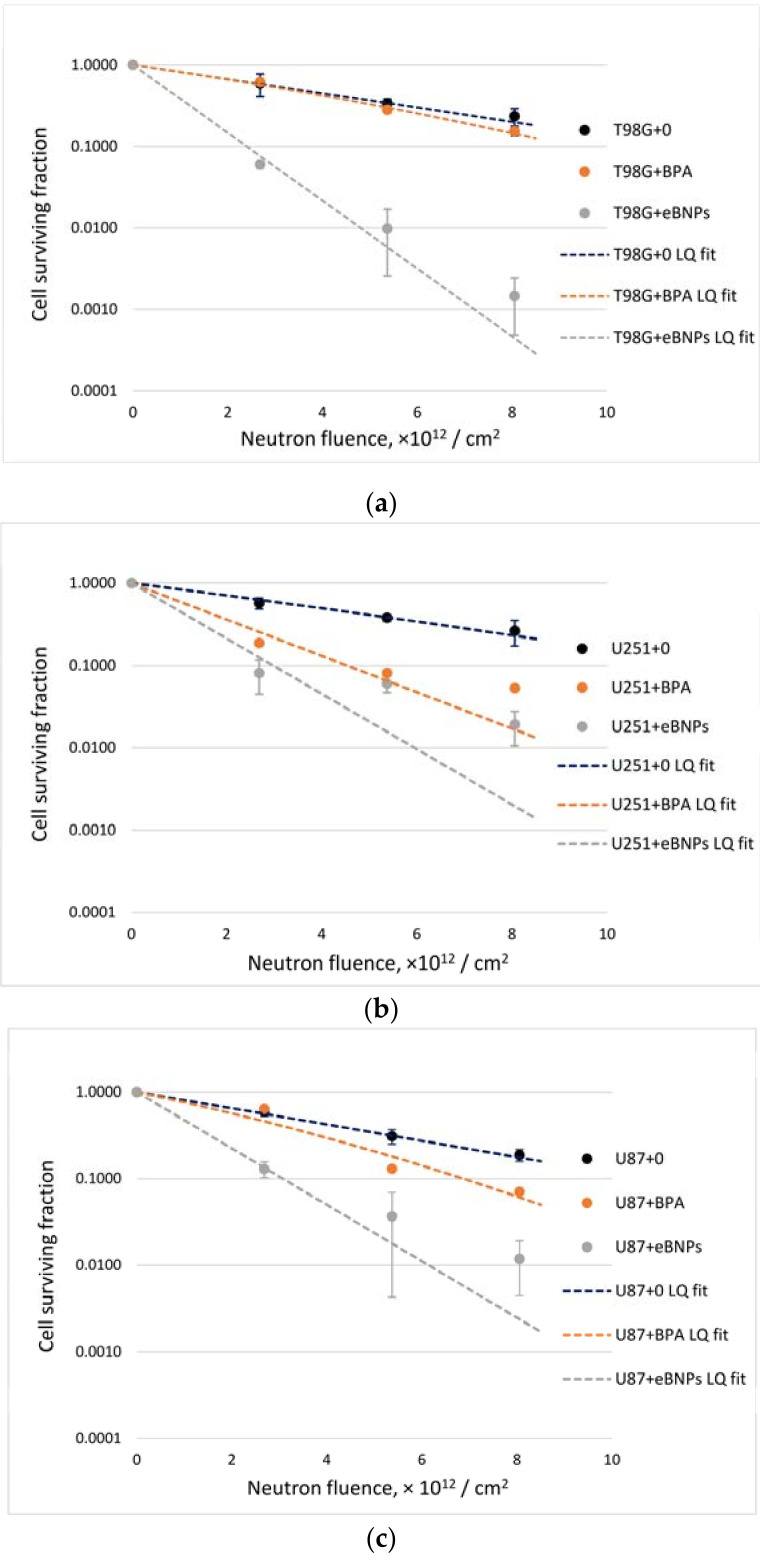
Cancer cell survival after BNCT. T98G (**a**), U251 (**b**) and U87 (**c**) cells were treated with eBNPs or BPA (40 µg ^10^B/mL), or left untreated (0), and irradiated with neutron fluences of 2.685, 5.370, or 8.055 × 10^12^/cm^2^. After dilution, seeding, and 14-day incubation, colonies of ≥50 cells were counted. The mean values of the cell survival fractions are plotted as curves with the error bars representing standard deviations.

Cell survival fractions after neutron beam irradiation with or without boron compounds and intergroup comparisons showed that the effect differed depending on the cell line and the compound (Figure 5, Table 2). Notably, eBNPs significantly reduced the ability of cells to proliferate within the whole fluence range in all cell lines compared to untreated, irradiated controls. This effect was more prominent in the T98G cells, where incubation with BPA had no significant influence on neutron beam irradiation (Figure 5a). In the U251 cells, both BPA and eBNPs induced a higher exponential decrease in cell survival compared to cells irradiated without boron (Figure 5b); however, the difference between both compounds was not significant at the point of 5.370 × 10^12^ neutrons/cm^2^ irradiation. In the U87 cells, the differences among all groups were significant except for BPA and the irradiated control at a point of 2.685 × 10^12^ neutrons/cm^2^ irradiation (Figure 5c).

Analysis of radiobiological parameters showed a significant difference between the neutron irradiation of cells with eBNPs and without boron (Table 3). The areas under the fitted curves (AUCs) and *F*_10_-values differed between the groups irradiated with BPA and without boron in the U251 and U87 cell lines, with no significant difference in the T98G cells. The difference between the radiobiological parameters in the eBNP and BPA groups was significant in the T98G and U87 cell lines and did not differ in the U251 cells.

## 4. Discussion

In this study, we used elemental boron nanoparticles derived from ^10^B microparticles in an aqueous solution and stabilized with 0.3% hydroxyethylcellulose as a boron compound for BNCT. The use of nanoparticles of this type with such a stabilizing agent had several goals. First of all, we aimed at showing the possibility of creating nanoparticles from elemental boron without impurities, bypassing complex multi-step chemical reactions, which is an advantage over previously known methods of creating boron nitride and boron carbide nanoparticles [27,31,32,33,34,35]. If only boron (^10^B) is used, its entire mass can be involved in the neutron capture reaction, which is critical for BNCT.

The dispersion of boron nanoparticles in an aqueous medium is characterized by low sedimentation stability over time; boron particles with a size of 1–12 nm are stable for 30–60 min after their synthesis (Table 1). Even with additional sonication and without further processing, the use of nanoparticles with size variations (Figure 3) may lead to different distribution and functions in biological systems when more homogeneous agents are preferable. Such particles form stable aggregates over a more extended period (30 days, Table 1) but are not feasible for biological experiments. Thus, the second goal was to solve the stability problem by choosing a stabilizer with low potential toxicity and good tolerance by biological systems. In terms of availability and safety, hydroxyethylcellulose was selected as a stabilizing agent to prevent aggregation. The use of a safe and non-toxic stabilizer allowed for the formation of a more homogeneous suspension of nanoparticle complexes with HEC in a colloidal solution (Table 1). When analyzing their effect on cell plating efficiency, these complexes showed low toxicity for cell cultures, both after 24 h of incubation with nanoparticles and fourteen days later (Figure 4). In T98G cells, eBNPs with HEC demonstrated lower toxicity than BPA (Figure 4b).

Considering solution viscosity and the convenience of its application in biological experiments, we empirically determined the most optimal concentration of 0.3% of hydroxyethylcellulose (4.5 mPa∙s). With this type of polymeric stabilizing matrix, the aggregative stability of boron nanoparticles was significantly increased over time; the particle size was maintained for more than three months (Table 1). The stability of eBNPs in HEC solution is preserved due to steric factors and non-covalent interactions of the polymer macromolecules with boron particles, namely the interaction of the dipoles of the polar groups of the macromolecules, the charged surface of boron particles, and dipole–surface interactions.

In addition, one of the goals of this study was to test the ability of nanoparticles accumulated in tumor cells to effectively reduce cell survival during BNCT in the absence of additional boron concentration in the medium, thereby overcoming the effect of low-molecular BPA washout from cells over time. We can assume that the effectiveness of the nanoparticles depended on the differences in the mechanisms of eBNPs and BPA accumulation and release by the cells. The exchange of BPA mainly depends on the ATB^0,+^, LAT1, and LAT2 amino acid transporters on the cell surface [63,64], and may also depend on the oxygen concentration [65], while nanoparticles are accumulated by endocytosis [28,29,30].

More complex boron-containing nanoparticles capable of penetrating the cell membrane have recently been developed for BNCT. Kaniowski et al., (2021) developed functional nanoparticles for the downregulation of the epidermal growth factor receptor (EGFR) oncogene based on composites of nucleic acids and C_2_B_10_H_12_ boron clusters [66]. The authors showed that the synthesized nanoparticles localized in the cell cytoplasm and reduced the expression of EGFR, as well as changed the phenotypes of the cells by reducing their cellular migration rate and causing growth arrest in the S-phase. The nanoparticles did not activate human macrophages while effectively penetrating human carcinoma A431 cells. This study is certainly valuable for further development of the method. However, the authors used boron clusters containing dozens of boron atoms, while we aimed to deliver thousands of boron atoms per particle.

Endocytosis can contribute to the accumulation of more boron with each nanoparticle captured by tumor cells over a unit of time compared to BPA regardless of the amino acid metabolism. Thus, the exchange of the drug for other amino acids from the extracellular space will ultimately provide the necessary tumor boron concentration. Since the presence of boron inside tumor cells in proximity to the nucleus is the most important for achieving the BNCT effect, the release of boron from the cells into the intercellular space, in the case of BPA, can lead to an insufficient neutron beam irradiation effect. In this regard, we tested a sample preparation method in which the cells were washed in PBS and placed in a fresh medium without boron for subsequent irradiation. Since irradiation requires a certain amount of time (the total time from placing the samples in a fresh medium to the end of irradiation with maximum fluence was about three hours), we assumed that the BPA would naturally leave the cells in exchange for other amino acids.

For comparison, we relied on data from our previously published experiments, where the cells were placed in the original boron concentration medium for irradiation to equalize the amount of boron inside and outside them and keep the boron compounds from leaking [44,58,61,67]. In these previous experiments, the goal was to test the effectiveness of BNCT using an accelerator-based neutron source with already-known boron compounds. In this study, with insight into the effectiveness of neutron irradiation at the accelerator, we tested a new drug with potentially new characteristics that differentiate it from BPA already at the in vitro level. Here, we showed the efficiency of boron nanoparticles to affect tumor cells in the presence of neutron irradiation regardless of the cell line (Figure 5, Table 2 and Table 3). We chose human glioma cell lines because invasive malignant brain tumors are one of the main targets for BNCT [68,69] and we have previously used these cultures in accelerator-based cell experiments, making direct comparisons of the present to previous results possible [44,61].

A new sample preparation technique, featuring cell washing prior to irradiation, showcased features of the interaction of both eBNPs and BPA with various cell cultures. In the case of T98G glioma, the colony-forming efficiency was significantly reduced when using nanoparticles and was tens or hundreds of times different from BPA or irradiation alone (Figure 5a, Table 2). The effect of BPA was not significantly different from neutron irradiation alone and was significantly lower than that of nanoparticles (Table 2 and Table 3). With identical irradiation, these results may, therefore, depend on two main conditions—the degree of boron accumulation and the rate at which it leaves the cells. Since we could not perform accumulation experiments (see study limitations), we can only assume that in the case of T98G cells, BPA either accumulated very poorly or left the cells very quickly. The nanoparticles, in turn, demonstrated significant efficiency, which may indicate both sufficient accumulation, creation of a therapeutic concentration (at least 10^9^ boron atoms per cell [20]), and maintenance of this concentration during the entire neutron irradiation.

As for U251 glioma cells, the results of irradiated samples with BPA and eBNPs differed significantly from the irradiated control, indicating the effectiveness of both drugs even when the cells were placed in a boron-free medium during irradiation (Figure 5b, Table 2). No differences between the nanoparticles and BPA were detected when the samples were analyzed at one of the three experimental points (Table 2, neutron fluence 5.370 × 10^12^/cm^2^). Additionally, when analyzing the radiobiological parameters and comparing the AUCs and *F*_10_ values, no significant differences were found between the eBNPs and BPA (Table 3), which may indicate both effective accumulation of both compounds and possible identical leaking from tumor cells while still maintaining an effective ^10^B concentration for the entire irradiation duration.

The U87 glioma cell line showed uniform, statistically significant differences between the three cell survival curves with eBNPs, BPA, and controls, respectively (Figure 5c, Table 2 and Table 3). Nanoparticles showed greater efficacy in reducing cell survival compared to BPA, providing either more efficient boron accumulation in the cells or no or minimal boron escape from the cells during irradiation. Nevertheless, even with a potential decrease in BPA concentration over time, the cells retained the necessary concentration of boron to achieve the BNCT effect.

As a result of these experiments, we showed that the BNCT effect when using both eBNPs and BPA might differ and depend on the chosen cancer cell type. In the case of BPA, placing the cells in blank medium before irradiation leads to a decrease or absence of the BNCT effect compared to the same cells irradiated in boron-containing medium [44,58,61,67]. The ability of eBNPs to effectively reduce cell colony formation after neutron irradiation was shown in all three lines, with a significant advantage of eBNPs over BPA in the case of the T98G and U87 cells (Figure 5, Table 2 and Table 3).

This study has certain limitations. One significant limitation is the lack of monitoring of the boron concentrations both in the cells and in the medium. Regarding BPA, we can be guided by previously obtained data, but experimental conditions, including irradiation settings, may differ. When using standard, previously effective methods of sample preparation and analysis of boron content by inductively coupled plasma atomic emission spectroscopy (ICP-AES [70]), we could not achieve complete dissolution of the stabilized boron nanoparticles in the acid medium. This may be due to the resistance of HEC to oxidation and the prevention of nanoparticle contact with the external environment, which may in turn, along with difficulties in determining the boron concentration, modulate the stability of synthesized complexes. This reduced or absent direct contact of boron with the components of biological systems could play a role in the observed low toxicity of the drug represented by the insignificant influence on cellular proliferation. This observed phenomenon might be used as additional proof of eBNP stabilization with HEC, which could only be shown by DLS analysis and comparison of non-stabilized and stabilized nanoparticles, initially leaving room for discussing another limitation of the study. A preliminary evaluation showed that it was possible to decompose HEC using microwave heating of samples (up to 250 °C) under high pressure (up to 25 atm), for example, using the Multiwave 7000 device (Anton Paar, Graz, Austria), which was not possible in this study due to logistical restrictions. Since it was impossible to determine the exact boron concentration in the samples, we could not accurately calculate the boron-dependent absorbed doses during the neutron irradiation, although the effect was comparable to our previous results [44,58,61,67]. Therefore, we relied on such accelerator performance indicators as the proton current milliampere-hour and calculated neutron fluence.

In our study, we analyzed eBNP toxicity only for tumor cells, leaving the question of toxicity for healthy cells as another limitation, which might still be critical for further BNCT application of the developed compound. There are several points to consider when discussing the toxicity of synthesized nanoparticles for healthy cells. The first point is the use of a non-toxic agent to stabilize eBNPs, hydroxyethylcellulose, which results in the low toxicity of the stabilized eBNPs for all cells, including healthy ones. The second important point is the further development of active tumor-targeting molecules on the surface of eBNPs to minimize their accumulation in healthy cells. At this stage, we can mainly rely on the low toxicity and biocompatibility of HEC, leaving the question of the healthy cell toxicity of eBNPs for further research.

It is known that the boron neutron capture reaction (^10^B(n,α)^7^Li) produces high-energy alpha particles and lithium nuclei, which directly affect cellular structures by transferring energy and leading to double-stranded breaks in DNA molecules that cause subsequent cell death [1,71]. Thus, the main component of BNCT can be described as high linear energy transfer (LET) irradiation. Despite the gamma component and fast neutrons, the effect of high LET alpha particles and Li nuclei can be considered the major factor. In our experiments, we used cells irradiated without boron as a control, theoretically removing from the equation the effect of nonlinear exposure to gamma irradiation (photons) and fast neutrons. Under these conditions, survival curves should be straight lines depending on the radiobiological parameter alpha, while the radiobiological parameter beta should be equal to zero [60]. However, when calculating these radiobiological parameters, we determined that the beta parameter was zero in only five out of nine sets of experiments (Table 3), while in the remaining four, it was non-zero but still significantly smaller than the alpha parameter. This may indicate the presence of additional conditions affecting the radiobiological parameters that changed the survival curves from straight lines into exponential curves (Figure 5). Due to a large number of irradiation components during BNCT and the associated mechanisms of tumor cell damage affecting the cell survival [71], it is probably impossible to exclude all the factors affecting the radiobiological parameters in our experiments. In their review, Maliszewska-Olejniczak et al., (2021) described DNA repair mechanisms during BNCT [71], which may also play a role in the radioresistance of tumor cells in our study and be responsible for the parameter beta difference from zero.

In previous experiments, we used a vertical neutron beam and horizontally placed samples [18,44,58,61,67], whereas, in this study, we used a horizontal neutron beam with vertical positioning of the samples (Figure 2). This may be more practical for clinical applications of BNCT, but it has certain limitations for cell experiments. In particular, this position of the samples makes it difficult to use a rotating stand, which can help unify the neutron flux to all samples. Consequently, when cell vials are in a static position, different samples can be exposed to different neutron irradiation, even if only slightly, and (to a greater extent) to differential effects of the gamma component of the beam and fast neutrons. This factor is the most plausible limitation compared to the differences in neutron fluence since neutrons should form a cloud in the area where the samples are located and most uniformly affect them based on the chosen beam-shaping assembly and the characteristics of the plexiglass phantom. Thus, future experiments should use a rotating stand to irradiate cell cultures when possible, which will contribute to more uniform irradiation of the samples and help to reduce errors in determining the radiobiological parameters [72].

The gamma component of the irradiation caused an absorbed dose of 2.997 Gy per 1 mAh (per 2.685 × 10^12^ neutrons/cm^2^), reaching a maximum of 8.991 Gy at three mAh (at 8.055 × 10^12^ neutrons/cm^2^). Such a dose by itself should have a significant effect on the cells. However, the calculations show that most of the photons had energies in the megavolt range (or close to it), while the main contributor to cell survival, in this case, should have been photons, with energies in the lower kilovolt range. In spite of this, such photons were small in number. Superficial X-rays (here 0.020–0.100 MeV), which could primarily affect the cells [48,57], accounted for 13.822%, indicating that the effect of photons was relatively insignificant compared to the results from the X-ray tube-based facilities working at lower energies [48]. This confirms the adequacy of our conditions for cellular experiments at this accelerator.

## 5. Conclusions

We synthesized novel elemental boron nanoparticles by an original method of ultrasonic dispersion/destruction of amorphous boron powder in an aqueous medium. We showed that the stabilization of heterogeneous nanoparticles with hydroxyethylcellulose leads to the unification of their fraction that remains unchanged over a long period of time. The stabilized nanoparticles showed no significant influence on cellular survival without neutron beam irradiation and significantly reduced the colony-forming capacity of all tested tumor cell lines after the irradiation. The BNCT effect using both nanoparticles and BPA can vary depending on the cell culture, with no effect possible in the case of BPA alone, when cells are placed in blank medium before irradiation. The results of our study support further tumor targeting-oriented modifications of these synthesized nanoparticles and subsequent in vivo BNCT experiments.

## 6. Patents

Uspenskij, S.A.; Khaptakhanova, P.A.; Zaboronok, A.A.; Kurkin, T.S.; Zelenetskij, A.N.; Selyanin, M.A.; Taskaev, S.Yu. METHOD OF PRODUCING A COMPOSITION FOR BORON NEUTRON CAPTURE THERAPY OF MALIGNANT TUMORS (VERSIONS). RU 2720458 C1. Application date: 6 June 2019. Registration date: 30 April 2020.

## Figures and Tables

**Table 1:** Stability of nanoparticle colloidal solutions.

Samples	Storage Time	ζ-Potential, mV	Size, nm
eBNPs in the aqueous medium	50 min	+6.8	1–12
	200 min	+34.3	200–350
	7 days	+36.1	210–390
eBNPs in the HEC solution	90 days	+45.6	25–28

**Table 2:** Cell surviving fractions (means ± SDs).

Samples		Neutron Fluence, ×10^12^ /cm^2^					
		2.685		5.370		8.055	
T98G	0	0.5912 ± 0.1828	^NS^	0.3354 ± 0.0424	^NS^	0.2328 ± 0.0558	^NS^
	BPA	0.6171 ± 0.0577	#	0.2807 ± 0.0245	#	0.1527 ± 0.0191	#
	eBNPs	0.0599 ± 0.0049	×	0.0098 ± 0.0072	#	0.0015 ± 0.0010	×
U251	0	0.5725 ± 0.0856	#	0.3800 ± 0.0368	#	0.2619 ± 0.0900	*
	BPA	0.1873 ± 0.0052	×	0.0804 ± 0.0022	^NS^	0.0530 ± 0.0028	×
	eBNPs	0.0806 ± 0.0358	#	0.0603 ± 0.0134	#	0.0191 ± 0.0086	×
U87	0	0.5978 ± 0.0650	^NS^	0.3091 ± 0.0672	×	0.1871 ± 0.0304	×
	BPA	0.6389 ± 0.0216	#	0.1302 ± 0.0071	*	0.0707 ± 0.0032	#
	eBNPs	0.1293 ± 0.0306	#	0.0367 ± 0.0369	×	0.0118 ± 0.0084	#

^NS^—not significant (*p >* 0.05); *—*p* < 0.05; ×—*p* ≤ 0.01; #—*p* ≤ 0.001 (intergroup comparison by one-way ANOVA; 0 versus BPA, BPA versus eBNPs, and eBNPs versus 0).

**Table 3:** Radiobiological parameters.

Samples	*α* (*alpha*)		*β* (*beta*)	Area under Curve	*p*-Values (AUC)	*F*_10_	*p*-Values (*F*_10_)
T98G	0	0.2013 ± 0.0424	0	4.0182 ± 0.4933	^NS^	11.7694 ± 2.3651	^NS^
	BPA	0.1926 ± 0.0346	0.0061 ± 0.0044	3.7831 ± 0.1943	#	9.3021 ± 0.4881	#
	eBNPs	0.9589 ± 0.0271	0	1.0429 ± 0.0297	#	2.4025 ± 0.0686	×
U251	0	0.1682 ± 0.0125	0.0018 ± 0.0031	4.3152 ± 0.3352	#	12.7821 ± 2.5917	×
	BPA	0.5083 ± 0.0072	0	1.9347 ± 0.0257	^NS^	4.5305 ± 0.0647	^NS^
	eBNPs	0.7738 ± 0.2347	0	1.3679 ± 0.4105	#	3.1690 ± 0.9710	#
U87	0	0.2153 ± 0.0310	0.0002 ± 0.0003	3.8313 ± 0.3441	*	10.7210 ± 1.4673	×
	BPA	0.2626 ± 0.0268	0.0106 ± 0.0039	2.9948 ± 0.0881	#	6.8663 ± 0.0893	#
	eBNPs	0.7501 ± 0.0874	0	1.3416 ± 0.1565	#	3.0982 ± 0.3674	×

All values are presented as means ± standard deviations, except for *p*-values. The *p*-value*s* ≤ 0.05 were considered indicative of statistical significance. ^NS^—not significant (*p* > 0.05); *—*p* < 0.05; ×—*p* ≤ 0.01; #—*p* ≤ 0.001 (intergroup comparison by one-way ANOVA; 0 versus BPA, BPA versus eBNPs, and eBNPs versus 0).

## Data Availability

The data presented in this study are available upon request from the corresponding author.
